# Guidelines for the Simulations of Nitroxide X-Band cw EPR Spectra from Site-Directed Spin Labeling Experiments Using S*imLabel*

**DOI:** 10.3390/molecules28031348

**Published:** 2023-01-31

**Authors:** Emilien Etienne, Annalisa Pierro, Ketty C. Tamburrini, Alessio Bonucci, Elisabetta Mileo, Marlène Martinho, Valérie Belle

**Affiliations:** 1 Aix Marseille University, CNRS (Centre National de la Recherche Scientifique), BIP (Bioénergétique et Ingénierie des Protéines), IMM (Institut de Microbiologie de la Méditerranée), 13009 Marseille, France; 2 Department of Chemistry, Konstanz Research School Chemical Biology, University of Konstanz, Universitätsstraße 10, 78457 Konstanz, Germany; 3 Aix Marseille University, CNRS (Centre National de la Recherche Scientifique), AFMB (Architecture et Fonction des Macromolécules Biologiques), 13009 Marseille, France; 4 Aix Marseille University, INRAE (Institut National de Recherche Pour L’agriculture, L’alimentation et L’Environnement), BBF (Biodiversité et Biotechnologies Fongiques), 13009 Marseille, France

**Keywords:** EPR, ESR, site-directed spin labeling, simulation, nitroxide, *EasySpin*, *SimLabel*

## Abstract

Site-directed spin labeling (SDSL) combined with continuous wave electron paramagnetic resonance (cw EPR) spectroscopy is a powerful technique to reveal, at the local level, the dynamics of structural transitions in proteins. Here, we consider SDSL-EPR based on the selective grafting of a nitroxide on the protein under study, followed by X-band cw EPR analysis. To extract valuable quantitative information from SDSL-EPR spectra and thus give a reliable interpretation on biological system dynamics, a numerical simulation of the spectra is required. However, regardless of the numerical tool chosen to perform such simulations, the number of parameters is often too high to provide unambiguous results. In this study, we have chosen *SimLabel* to perform such simulations. *SimLabel* is a graphical user interface (GUI) of *Matlab*, using some functions of *Easyspin*. An exhaustive review of the parameters used in this GUI has enabled to define the adjustable parameters during the simulation fitting and to fix the others prior to the simulation fitting. Among them, some are set once and for all (g_y_, g_z_) and others are determined (A_z_, g_x_) thanks to a supplementary X-band spectrum recorded on a frozen solution. Finally, we propose guidelines to perform the simulation of X-band cw-EPR spectra of nitroxide labeled proteins at room temperature, with no need of uncommon higher frequency spectrometry and with the minimal number of variable parameters.

## 1. Introduction

Site-directed spin labeling (SDSL) combined with electron paramagnetic resonance (EPR) spectroscopy is a powerful method for investigating the dynamics of conformational changes in biological macromolecules such as proteins [1,2,3,4,5]. This method is based on the selective grafting of paramagnetic labels at selected sites and on its analysis by EPR spectroscopy. The spin labels are usually functionalized to react specifically with cysteine residues, often introduced by site-directed mutagenesis. Alternatively, tyrosine residues [6,7,8] and unnatural amino acids [9,10,11,12] can also be targeted, in particular when cysteine residues are involved in protein function or structure. Among spin labels currently available, nitroxides show key spectroscopic features. Indeed, the power of nitroxide-based SDSL combined with continuous wave (cw) EPR relies on the sensitivity of the nitroxide EPR spectral shape to the mobility of the label in the nanosecond time window. As this mobility is related to the local environment that surrounds the spin label, information can be obtained on the secondary and tertiary structures of the labeled protein under study.

Nitroxide spin labels are highly stable radicals. In the case of gem-dimethyl nitroxide labels, they exhibit typical three-line cw EPR spectra resulting from the interaction of the electron spin (S = 1/2) with the ^14^N nuclear spin (I = 1). The associated magnetic tensors, **g** and **A** (^14^N hyperfine coupling), are anisotropic. These tensors are considered diagonal in the nitroxide frame where the *x*-axis and the *z*-axis are defined along the N-O bond direction and along the 2p_z_-orbital of the nitrogen atom, respectively. This anisotropy explains the sensitivity of the cw EPR signal to the mobility of the label. A reduced mobility of the label results in a partial motional averaging of the anisotropy, giving a broadened spectrum. On the other hand, a high mobility of the label implies a large motional averaging of the anisotropy resulting in a narrow cw EPR signal, driven by the averaged components of magnetic tensors g_iso_ and A_iso_.

Most of the time, the experimental SDSL–cw EPR spectra contain multiple components which originate from the coexistence of multiple conformation of the protein and/or different rotamers of the spin label [13,14,15]. These spectra are then a linear combination of individual spectra. To extract the components and quantify their dynamics, a numerical simulation is required. One method is to use the *Matlab* toolbox *EasySpin* [16] and *SimLabel* [17], a graphical user interface (GUI) we have previously developed especially for such simulations.

In *SimLabel*, each component is described by the eigenvalues of the magnetic tensors: g_x_, g_y_ and g_z_ for the **g**-tensor and A_x_, A_y_, A_z_ for the **A**-tensor, associated to a nuclear spin (I = 1 for ^14^N nitroxide). A rotational correlation time t_corr_, isotropic or anisotropic, characterizes the reorientational motion of the spin label [18]. An additional Voigtian convolutional broadening, parametrized by Gaussian and Lorentzian full widths at half maximum, is available. Finally, in the case of a multicomponent simulation, a relative weight of the considered component is added. Consequently, to simulate the spectrum of a single component in *SimLabel*, at least 12 parameters have to be tuned. This high number of parameters, especially in the case of multicomponent spectra, makes their unambiguous determination very difficult.

One way to overcome this difficulty is to determine the magnetic parameters thanks to high field cw EPR experiments (W band and higher frequency) [19,20], or multifrequency experiments (X-band and at least W-band) [21,22,23]. However, the low number of facilities having high frequency spectrometers makes this approach not accessible to most EPR users.

The aim of this paper is to propose guidelines to perform reliable simulations of X-band spectra of nitroxide at room temperature with as much confidence as possible for the quantitative study of protein dynamics. To this purpose, we will review all the parameters used in *SimLabel.* We will show the spectral influence of some of them and, to reduce the number of adjustable parameters in the fitting procedure or to limit their range of variation, we will establish constraints for the others. Finally, we will apply this procedure to simulate the spectra of two labeled proteins chosen as examples.

## 2. Results and Discussion

Extracting quantitative information from the cw EPR spectrum of a nitroxide-labeled protein recorded at room temperature consists in determining the number of components, their weights and their rotational correlation times. Even if the remaining parameters are not strictly related to dynamics, their value should not be overlooked to ensure the relevance of the simulation. For this reason, we present here an exhaustive review of all the parameters used by *SimLabel* to simulate cw X-band spectra of nitroxides grafted on macromolecules.

Before starting, it is worth specifying that the experimental spectra must be recorded in optimal conditions (no saturation and no overmodulation). Moreover, we highly recommend calibrating the experimental magnetic field using as a reference a sample with a known g-value. The absence of calibration could lead to wrong simulated g values that will result in erroneous values for the other parameters (see Section 2.1.2).

### 2.1. Review of the Parameters Used by SimLabel

#### 2.1.1. Magnetic Tensors g and A

In *SimLabel*, the magnetic properties of nitroxide spin labels are described by the eigenvalues of the anisotropic **g**-tensor and **A**-tensor (given in mT). The principal axis of these tensors is taken parallel and aligned with the molecular frame of the nitroxide. Previous high field studies have shown that g_x_ and A_z_ are the most sensitive indicators of the micro-environment polarity and proticity, due to the electric field produced along the NO-bond direction (*x*-axis) and hydrogen bond formation [22,24,25].

Concerning the g values, as g_y_ and g_z_ are less sensitive to the local surrounding, they can be set to values found in the literature from high field analysis. We propose to take g_y_ = 2.0061 and g_z_ = 2.0022 as fixed values [25,26]. On the other hand, a linear correlation between g_x_ and A_z_ has been previously reported for nitroxide grafted on proteins, based on a series of high frequency EPR spectra recorded at low temperature [20,25]. This correlation can be expressed as

g_x_ = f(A_z_) = −0.0025 × A_z_ + 2.0175 (1)

where A_z_ is in mT. Practically, the reported experimental g_x_ values of these studies followed this correlation within ±0.0002. However, it is easy to check with *SimLabel* that such a variation of g_x_ has no effect on the nitroxide X-band spectrum whatever the correlation time is. Then we propose to calculate and set g_x_ from Equation (1) when A_z_ is known.

**Figure 1:**
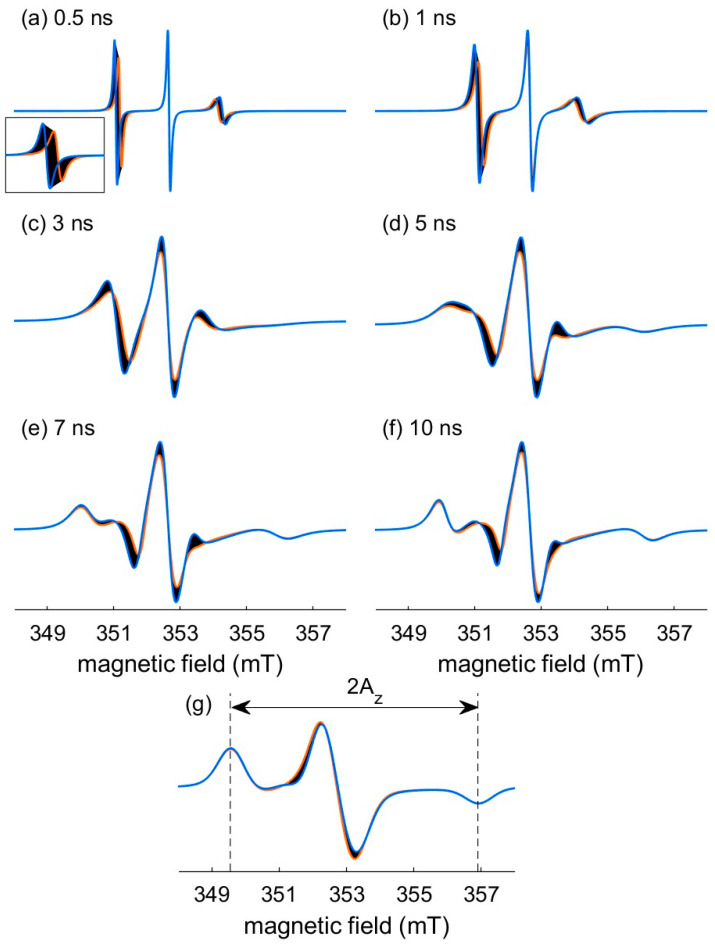
Influence of variations in A_xy_ (0.40 mT ≤ A_xy_ ≤ 0.60 mT) on the nitroxide X-band cw EPR spectral shape for: (**a**) t_corr_ = 0.5 ns; (**b**) t_corr_ = 1 ns; (**c**) t_corr_ = 3 ns; (**d**) t_corr_ = 5 ns; (**e**) t_corr_ = 7 ns; (**f**) t_corr_ = 10 ns; (**g**) frozen solution. Each panel is the superposition of 100 simulated spectra (black traces) with A_xy_ ranging from 0.40 mT to 0.60 mT. The limit spectra obtained with A_xy_ = 0.60 mT = 16.4 MHz and A_xy_ = 0.40 mT = 11.2 MHz are colored (blue trace and orange trace, respectively). Inset in panel (**a**): zoom of the low field line. All the spectra are normalized to their integrated intensity. Parameters for the simulations: g_x_ = 2.0084, g_y_ = 2.0061, g_z_ = 2.0022, A_z_ = 3.66 mT = 102.6 MHz, at 9.9 GHz, no additional broadening.

The **A**-tensor of nitroxide has almost axial symmetry: A_x_ = A_y_ = A_xy_ ≠ A_z_, where A_z_ has the largest value. A particular feature of X-band nitroxide spectra in a frozen solution is that the outer lines are separated by 2A_z_ (Figure 1g). Then, recording the EPR spectrum of the same sample in frozen conditions is a way to experimentally measure A_z_ without ambiguity. Concerning A_xy_, its value typically ranges from 0.40 mT (11.2 MHz) to 0.60 mT (16.4 MHz) [23,25]. To evaluate the effect of this parameter on the EPR spectral shape, Figure 1a–f shows the influence of A_xy_ variation in this range for different correlation times t_corr_. The dark areas emphasize this influence. The orange and blue traces stand for the minimal and the maximal boundaries for A_xy_, respectively. An effect on the position and amplitude of the lines can be observed. In the fast regime (see Section 2.1.2., Figure 1a,b), only the outer lines are affected whereas only the central lines are concerned in the slow regime (see Section 2.1.2., Figure 1e,f). Note that, in the fast regime, the splitting of the lines is determined by A_iso_ and, even if A_z_ is set, A_iso_ is modified when A_xy_ is varying.

Finally, these considerations lead to reduce the number of variable magnetic parameters from six (g_x_, g_y_, g_z_, A_x_ A_y_ and A_z_) to one in a constrained range (0.40 ≤ A_xy_ ≤ 0.60 mT). A_z_ is measured on the spectrum of the frozen solution, g_x_ is calculated from Equation (1), g_y_ = 2.0061 and g_z_ = 2.0022 are fixed. Note that, even if the A_xy_ range of variation is weak, allowing this variation enables to adjust the position and amplitude of the lines.

#### 2.1.2. The Rotational Correlation Time

The cw EPR spectral shape of a nitroxide labeled protein directly reflects the rotational motion of the nitroxide label and, consequently, that of its local microenvironment. This motion is characterized by the rotational correlation time t_corr_. This parameter includes contributions of the side-chain internal motions, backbone fluctuations and the overall tumbling of the protein [27]. X-band EPR spectra of nitroxide can probe motions on timescales between 0.1 and 20 ns. Three regimes of motion associated to the nitroxide spectral shape can be described [28]: fast (Figure 1a,b), intermediate (Figure 1c,d) and slow (Figure 1e,f). Note that these motional regimes result from the X-band EPR time scale. For higher frequency, as W-band for instance, the nitroxide spectrum for a given t_corr_ shows a less complete averaging of the anisotropies with respect to X-band. As a result, spectra detected at higher frequency are more sensitive to faster motions, whereas spectra detected at lower frequency are more sensitive to slower motions [26].

X-band spectra in the fast motion regime (t_corr_ < 1ns) exhibit three narrow lines (peak-to-peak width less than 0.2 mT). These spectra result from the almost complete averaging of the **A**-tensor anisotropy, as well as **g**-tensor. The position of the central line and the splitting of the lines are driven by g_iso_ and A_iso_, respectively. On the other hand, the spectra in the slow-motion regime exhibit broad lines, which amplitudes and positions are sensitive to the anisotropy of **A**-tensor and **g**-tensor.

**Figure 2:**
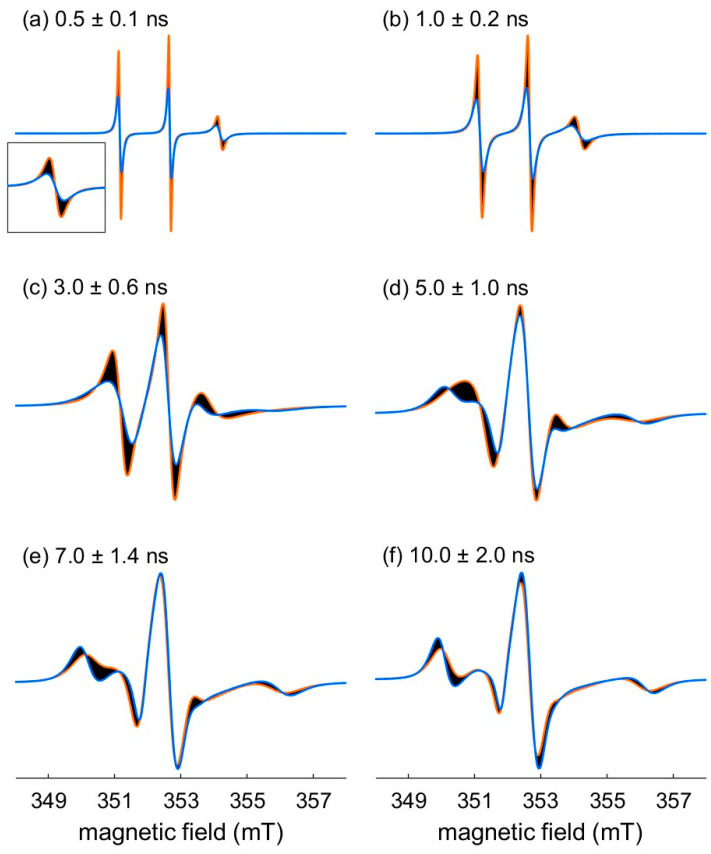
Influence of a relative variation (20%) of t_corr_ on the nitroxide X-band cw EPR spectral shape for: (**a**) t_corr_ = 0.5 ns; (**b**) t_corr_ = 1 ns; (**c**) t_corr_ = 3 ns; (**d**) t_corr_ = 5 ns; (**e**) t_corr_ = 7 ns; (**f**) t_corr_ = 10 ns. Each panel is the superosition of 100 simulated spectra (black traces) with a correlation time ranging from 0.8*t_corr_ to 1.2*t_corr_. The traces for the minimal and the maximal considered t_corr_ are orange and blue, respectively. Inset in panel (**a**): zoom of the low field line. All the spectra are normalized to their integrated intensity. Parameters for the simulations: g_x_ = 2.0084, g_y_ = 2.0061, g_z_ = 2.0022, A_xy_ = 0.45 mT = 12.6 MHz, A_z_ = 3.66 mT = 102.6 MHz, at 9.9 GHz, no additional broadening.

As t_corr_ is crucial to characterize the dynamics of a spin label, we may wonder what the influence of this parameter on the spectral shape could be. One way to address this question is to visualize the effect on the spectral shape by varying the t_corr_ value within a range of 20%. Figure 2 shows the influence on the spectral shape for different time scales of t_corr_ within a 20% variation. The dark areas emphasize this influence. The orange and blue traces stand for the minimal and the maximal considered t_corr_, respectively. We can appreciate significant effects of this variation on the spectral amplitudes and linewidths. The same relative variation of t_corr_ (20%) has a greater impact on the spectra shown Figure 2a–c, where the linewidths are strongly affected. On the other hand, on the last three spectra (Figure 2d–f), the position and width of the outer lines are also affected but to a lesser extent.

**Figure 3:**
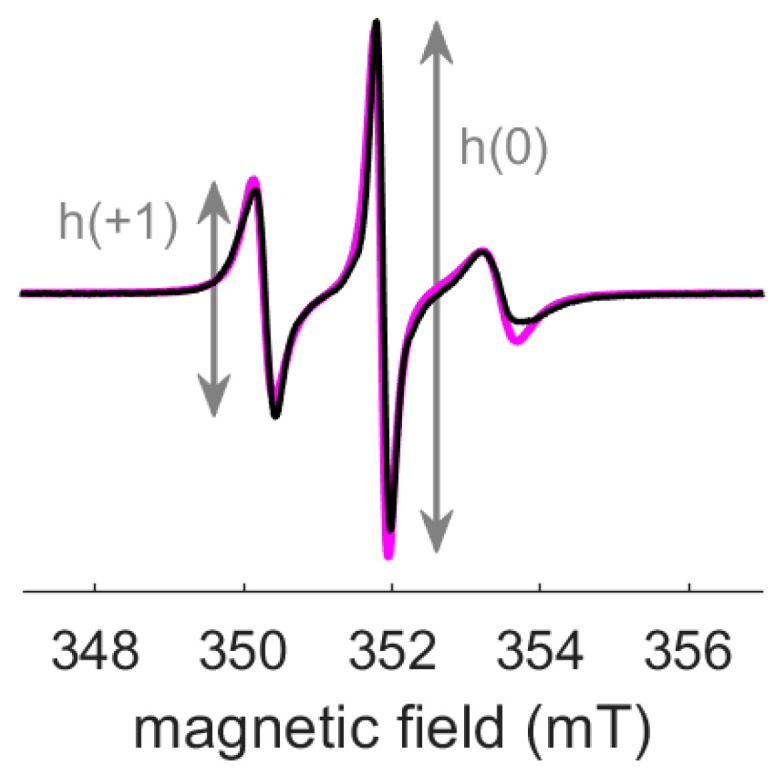
Experimental X-band cw EPR spectrum of nitroxide labeled protein (black trace) and its simulation with an axial t_corr_ (magenta trace). This spectrum is characterized by h(0)/h(+1) ≈ 2, where h(0) and h(+1) are the amplitude of the central and low field lines, respectively. Parameters for the simulation: t_corr,xz_ = 4.5 ns, t_corr,y_ = 0.2 ns, g_x_ = 2.0082, g_y_ = 2.0061, g_z_ = 2.0022, A_xy_ = 0.42 mT = 11.8 MHz, A_z_ = 3.70 mT = 103.7 MHz, at 9.9 GHz, no additional broadening.

In further consideration of rotational correlation times, we sometime observe that experimental spectra with narrow lines cannot be reproduced with an isotropic correlation time, even if two components are considered. Figure 3 shows such an experimental spectrum and its simulation. The amplitude of the central line h(0) is around twice the amplitude of the low field line h(+1). Considering an anisotropic correlation time, with a long t_corr_ along x and z directions and a short t_corr_ along the y direction, enables to better reproduce this typical shape. In order to minimize the number of parameters, anisotropic correlation time can firstly be considered as axial (only one extra parameter with respect to isotropic t_corr_), as presented in [23].

#### 2.1.3. Linewidths and Additional Broadening

An experimental cw EPR spectrum of a motional spin label exhibits a microwave frequency dependent linewidth that has multiple origins. Firstly, this linewidth is proportional to t_corr_ [29], as modeled by the *chili* function of *EasySpin* used in the slow motion mode of *SimLabel*. Regardless of the dynamic aspects, the spin label relaxation and then the experimental linewidth can also be affected by the presence of relaxing agent in the surrounding. This property is well known and has been exploited to investigate nitroxide accessibility by power saturation method [30]. Multiple factors can broaden the experimental linewidth, such as unresolved hyperfine couplings or dipolar interactions. Therefore, we can separate the origins of the experimental linewidths into two types, the first one being related to the dynamics and the second one not.

In the *slow motion* mode of *SimLabel*, the linewidths related to dynamics result from the computation of the *chili* function. To model a potential broadening not related to dynamics, an additional line broadening is available for the simulated spectrum. This broadening, where no physical model is assumed, consists in an isotropic convolution of the spectrum with either a pure Gaussian profile, or a pure Lorentzian profile or a Voigtian profile (convolution of Gaussian profile with Lorentzian profile). Once again, in order to minimize the number of parameters, we chose to consider only Lorentzian broadening, characterized by its full width at half maximum lw_lor_ given in mT.

**Figure 4:**
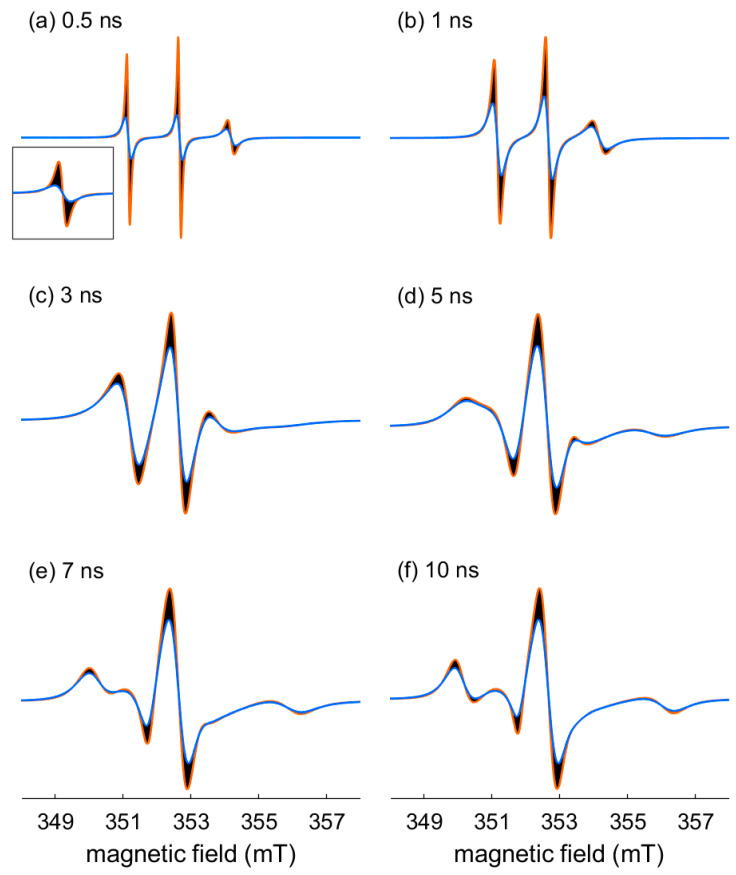
Influence of the variations of the additional Lorentzian broadening (0 ≤ lw_lor_ ≤ 0.15 mT) on the nitroxide X-band cw EPR spectral shape for: (**a**) t_corr_ = 0.5 ns; (**b**) t_corr_ = 1 ns; (**c**) t_corr_ = 3 ns; (**d**) t_corr_ = 5 ns; (**e**) t_corr_ = 7 ns; (**f**) t_corr_ = 10 ns. Each panel is the superposition of 100 simulated spectra (black traces) with lw_lor_ varying from 0 to 0.15 mT. The spectra obtained with no broadening (lw_lor_ = 0) and lw_lor_ = 0.15 mT are colored (orange and blue trace, respectively). Inset in panel (**a**): zoom of the low field line. All the spectra are normalized to their integrated intensity. Parameters for the simulations: g_x_ = 2.0084, g_y_ = 2.0061, g_z_ = 2.0022, A_xy_ = 0.45 mT = 12.6 MHz, A_z_ = 3.66 mT = 102.6 MHz, at 9.9 GHz.

In our experience, the typical encountered lw_lor_ range from 0 mT (no broadening) to 0.15 mT. Figure 4 shows the influence of this parameter within this range on the spectral shape for different t_corr_. The dark areas give an overview of the extent of the parameter variation. The orange and blue traces stand for the minimal and the maximal considered lw_lor_, respectively. We can notice an effect of lw_lor_ variation on the spectral amplitudes and linewidths. As expected, this effect is more significant for the spectra with narrow lines.

#### 2.1.4. Multicomponent Spectra

So far, we considered spectra arising from only one single spectral component. However, experimental spectra on labeled proteins mainly contain multiple spectral components characterized by different dynamics. If the remaining free nitroxide labels are properly fully removed from the sample and only one type of labeled protein is present in the sample, these components arise from different rotamers of the labels or conformers of the proteins [13,14,15]. In the following, we will only consider this last case. The spectrum arising from such a sample is a linear combination of single component spectra. A new parameter must be therefore introduced for each component in *SimLabel*: the weight. We previously saw the influence of variations in A_xy_, t_corr_, and lw_lor_ (Figure 1, Figure 2 and Figure 4) on the spectral shape of a single component. As previously said, those variations result, in particular, in changes in the amplitude of the spectral lines. Consequently, in the case of multicomponent spectra, the weights of each component could be strongly affected by erroneous determination of t_corr_, lw_lor_, and A_xy_.

Concerning the magnetic parameters, an interesting example can be found in the literature [23]. The authors produced two samples. The first sample contains a nitroxide labeled protein giving a cw EPR spectrum with a single fast component. The second one contains the same labeled protein interacting with a physiological partner. The cw EPR spectrum showed a mixture of two components: a fast component (unbound state) and a slow one (bound state). Interestingly, these two samples gave undistinguishable W-band spectra at low temperature. Thus, in the simulations, the authors used the same magnetic parameters whatever the component. Therefore, we propose to extrapolate this result in the following and consider the same magnetic parameters for all the components of a given labeled protein, whatever their dynamics.

The last parameter to consider for running simulations with *SimLabel* is the additional broadening. We have seen that this additional broadening does not depend on any dynamic aspect. Consequently, we assume in the following that, if an additional broadening is required for one component, especially in the fast motion regime, this broadening might be applied to all the components, whatever their dynamics.

Thus, although introducing new component(s) in a simulation will automatically increase the number of parameters, the previous considerations prevent this number from being proportional to the number of components. Each component is described with a specific correlation time and a weight, although the magnetic parameters and the potential additional broadening are common to all components. Note that *SimLabel* automatically offers the possibility to consider this similarity of components (same magnetic parameters and if present, same broadening) for automatic fitting when the parameters of the components differ only in the rotational correlation time.

### 2.2. Simulation of Experimental Spectra with SimLabel

In light of the previous considerations, we will present guidelines to simulate the X-band spectra of nitroxide labeled proteins recorded at room temperature. These guidelines include what should be done before and during the simulation. Finally, two examples of application will be presented.

#### 2.2.1. Requirements before the Simulation

Regardless the sample, before simulating the data, two independent steps have to be carried out: (i) determine A_z_ and calculate g_x_, and (ii) calibrate the magnetic field.

As specified before, determining A_z_ requires to record a spectrum of the sample under investigation in frozen state (115 K achieved with liquid nitrogen for instance). 2A_z_ is directly extracted from this spectrum with *SimLabel* by measuring the splitting between the outer lines in mT (Figure 1g). g_x_ is then deduced from the linear correlation given by Equation (1): g_x_ = f(A_z_). Note that this correlation is integrated in the last version of *SimLabel* and the assignment of f(A_z_) to g_x_ is automated. As g_y_ and g_z_ are set to 2.0061 and 2.0022, respectively, at this point, only A_xy_ is undetermined among the magnetic parameters, and a default value of 0.50 mT can be considered as a starting point.

Concerning the magnetic field calibration, this can either be performed with a standard sample of known g value or by using a so-called g-marker (see Section 3.3). In this case, as the g-marker signal is superimposed on the signal of interest, two spectra are successively recorded: one with the g-marker in (for field calibration purpose) and one with the g-marker out (for simulation).

#### 2.2.2. Recommendations for the Simulation Using *SimLabel*

First of all, note that, in the following, adjusting a parameter or modifying a parameter means that the user changes this parameter “manually” (no automatic fitting), thanks to the sliders in the *Real Time* mode for instance. The adjustment is achieved when the resulting simulation visually fits well the experimental spectrum. For weak effect, the *fit indicator* parameter of *SimLabel* can be used (see *SimLabel* documentation). The best simulation is the closest to zero for this parameter and we suggest that, below the value of 0.02, the simulation can be considered as satisfactory.

In general, whatever the type of simulation, the complexity of the model used increases as the procedure progresses. This is accompanied by an increase in the number of adjustable parameters used to describe the system. However, as a large increase in the number of parameters will always result in a good simulation, it is important to be careful not to excessively increase this number.

As far as simulations of X-band cw-EPR spectra of labeled proteins are concerned, determining a priori the number of components to include in the simulation is not an easy task. It is advised to start with one single component and then increase the complexity of the system until achieving a satisfactory simulation. The simplest model, used as a starting point, is a single component, with an isotropic rotational correlation time and no supplementary broadening. Following the considerations resulting from the review in Section 2.1, only two parameters (t_corr_ and A_xy_) have to be adjusted for the simulation to fit the experimental spectrum. If no acceptable simulation can be found, we suggest to introduce and adjust lw_lor_ (one supplementary parameter) or axial anisotropic t_corr_ (t_corr,xz_ and t_corr,y_ instead of t_corr_, hence an increase of one unit in the number of parameters) or both (increase of 2 units in the number of parameters). In the same way, if no satisfactory simulation can be found, two components, with the same magnetic parameters, should be considered, both with an isotropic rotational correlation time and with no supplementary broadening. If adjusting the five parameters (t_corr_ and weight for both components, and A_xy_) does not give a satisfactory result, the model could be made more complex, step by step, as presented above for one single component, keeping in mind that if a supplementary broadening is required, it should be applied to all components.

Finally, despite the required cautions, it may happen that a small number of unreacted labels (not covalently bound to the protein) is present in solution and contributes to the spectrum with a very narrow signal. In that case, a few percent of a supplementary fast component can be added to the simulation with its own g_iso_ and A_iso_.

#### 2.2.3. Application to Sample 1

**Figure 5:**
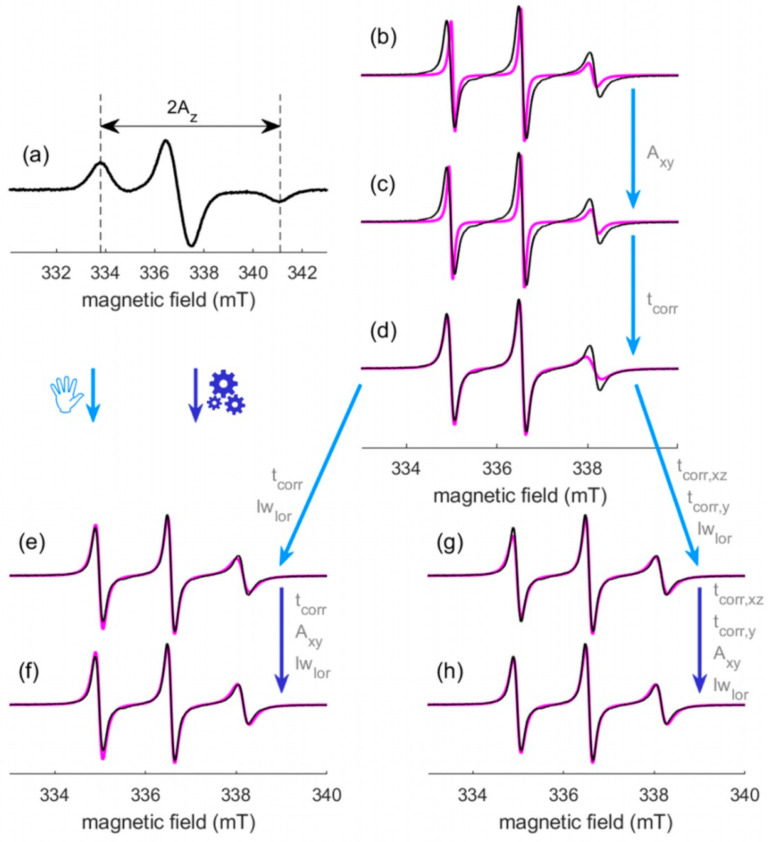
Different steps during the simulation and fitting process for sample 1. (**a**) X-Band spectrum of the frozen solution (115 K) enables to extract 2A_z_ from the position of the two outer lines (A_z_ = 3.64 mT). (**b**–**h**) Superpositions of the experimental spectrum (black traces) recorded at room temperature with a calibrated magnetic field and the different simulations (magenta traces) of the fitting procedure. The simple arrows indicate the sequence of intermediate simulations. Light and dark blue arrows stand for manual and automatic fitting, respectively. The adjusted parameters are indicated in grey. The parameters of these simulations are given Table 1.

Sample 1 contains a nitroxide labeled model protein. Details on this sample are given in Section 3.1.1. Three cw EPR spectra of sample 1 were recorded: one spectrum at 115 K to determine A_z_ = 3.64 mT (Figure 5a) and two spectra at room temperature with the g-marker in and out. Once the magnetic field is calibrated, one can start the simulation of the spectrum with the g-marker out (black trace of Figure 5b–h).

One single component is considered to start, with an isotropic rotational correlation time and no supplementary broadening. A_z_ is set to 3.64 mT and a default value of A_xy_ = 0.50 mT is first considered. The g values are also set: g_x_ = f(A_z_) = 2.0084, g_y_ = 2.0061 and g_z_ = 2.0022. A first simulation with t_corr_ = 0.5 ns for example is run (Figure 5b and Table 1b).

The splitting between the outer lines, driven by A_iso_ in this mobility range, appears to be too small and needs to be increased. Adjusting A_xy_ enables to modify A_iso_ and to adjust the outer lines to the correct positions (Figure 5c and Table 1c). The next step consists in modifying the correlation time. Unfortunately, no t_corr_ can correctly reproduce the shape of all three lines (Figure 5d and Table 1d), and a supplementary Lorentzian broadening is introduced. Both t_corr_ and lw_lor_ are then modified to fit the experimental spectrum (Figure 5e and Table 1e). Although a good simulation is obtained as confirmed by the *fit indicator* value, an automatic fitting (see Section 3.4) is run for a last improvement of the three parameters t_corr_, A_xy_ and lw_lor_ (Figure 5f and Table 1f). Surprisingly, the simulation provided by *esfit* (see Section 3.4.) gives a *fit indicator* calculated by *SimLabel,* which is slightly worse than the *fit indicator* of the initial simulation (Figure 5e and Table 1e). This difference can be explained by different normalizations of spectra considered in *esfit* and in *SimLabel*.

As the best simulations are not the same for *esfit* and *SimLabel*, we can try an axial t_corr_ from step (d). Once again, no convenient simulation can be obtained without a supplementary broadening. Introducing lw_lor_ in addition to axial t_corr_ and adjusting these parameters allow obtaining the spectrum of Figure 5g (Table 1g). As previously, we finally run an automatic fitting to fine tune the four parameters t_corr,xz_, t_corr,y_, A_xy_ and lw_lor_ (Figure 5h and Table 1h). In that case, the simulation provided by *esfit* gives the best *fit indicator* in *SimLabel* and this final simulation should be chosen for the interpretations.

#### 2.2.4. Application to Sample 2

**Figure 6:**
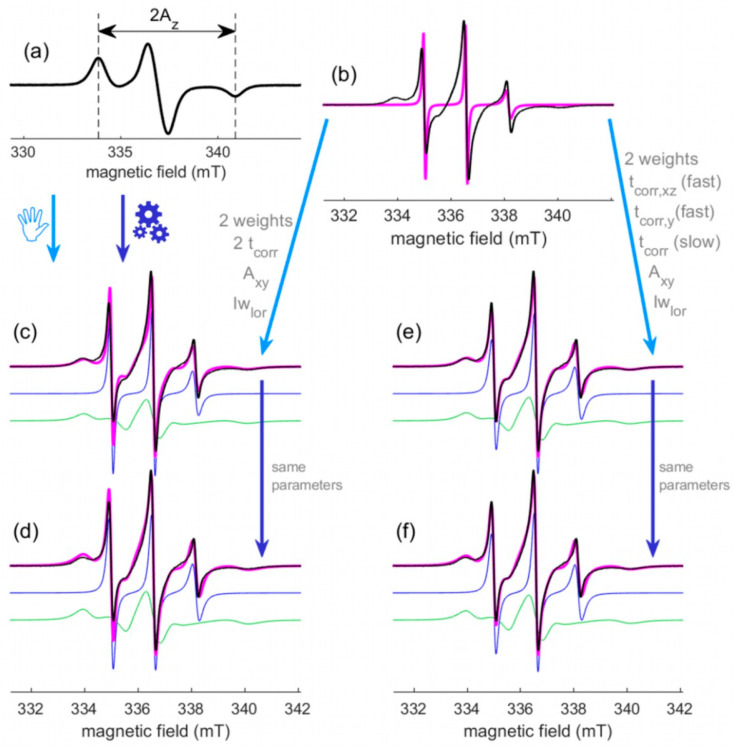
Different steps during the simulation and fitting process for sample 2. (**a**) The experimental spectrum of the frozen solution (115K) to extract 2A_z_ from the positions of the two outer lines (A_z_ = 3.54 mT). (**b**–**f**) Superpositions of the experimental spectrum (black traces) recorded at room temperature with a calibrated magnetic field and the different simulations (magenta traces) of the fitting procedure. The simulations in (**c**–**f**) result from the sum of the spectra of a fast component (blue traces) and a slow component (green traces). The simple arrows indicate the sequence of intermediate simulations. Light and dark blue arrows stand for manual and automatic fitting, respectively. The adjusted parameters are indicated in grey. The parameters of these simulations are given Table 2.

Sample 2 is a nitroxide labeled protein described in Section 3.1.2. This sample was chosen as a typical example of multicomponent spectra. As for sample 1, three spectra were recorded. The spectrum of the frozen solution provides A_z_ = 3.54 mT (Figure 6a). The two spectra at room temperature (g-marker in and out) provide the spectrum to be simulated with no marker in and with a calibrated magnetic field (black trace of Figure 6b–f).

Applying the procedure described for sample 1 with a single component does obviously not lead to an acceptable simulation, because this signal is clearly made of two components: a narrow one (short t_corr_) and a broad one (long t_corr_). Anyway, the position of the outer lines of the narrow signal can be adjusted thanks to A_xy_ of a single fast component (Figure 6b and Table 2b). This value will be used in the next step with two components (isotropic t_corr_ and same magnetic parameters): the so-called fast component with the narrowest signal (blue spectrum in Figure 6c–f) and the so-called slow component with the broadest one (green spectrum in Figure 6c–f). When we play with t_corr_ and the weight of these two components, we realize that fitting the experimental spectrum is not possible with this model and its complexity should be increased.

A supplementary Lorentzian broadening is included for the two components. The variable parameters, indicated along the light blue arrow, are then modified to fit the experimental spectrum (Figure 6c and Table 2c). Finally, an automatic fitting (see Section 3.4) is run to fine tune these six parameters: t_corr_ and weight of the two components, A_xy_ and lw_lor_ (Figure 6d and Table 2d).

The amplitude of the simulated narrow line at low field can be seen as too high in comparison to the experimental one. An axial t_corr_ for this component (fast) is then introduced to change this amplitude ratio. As previously considered from step (b) to (c), playing with the adjustable parameters leads to the introduction of a supplementary broadening. A simulation can then be found by adjusting the seven variable parameters: t_corr,xz_, t_corr,y_ for the fast component, t_corr_ for the slow component, their two respective weights, A_xy_ and lw_lor_. (Figure 6e and Table 2e). Finally, an automatic fitting is run to fine tune these parameters (Figure 6f and Table 2f), providing a better agreement for all the lines and the best *fit indicator* of all the simulations. This last simulation should be chosen for the interpretations.

## 3. Materials and Methods

### 3.1. Origin of the Samples

#### 3.1.1. Sample 1

NarJ (27 kDa) is a cytosolic chaperone from Escherichia coli ensuring folding and assembly of the membrane-bound-respiratory nitrate reductase complex, NarGHI, member of a large group of molybdenum-containing enzymes. NarJ contains one natural occurring Cysteine residue (Cys 207) located in the C-terminal region. NarJ was purified and labeled with the Maleimido-Proxyl nitroxide (Sigma-Aldrich, St. Louis, MO, USA) as described in [31].

#### 3.1.2. Sample 2

Sample 2: UreG from Sporosarcina pasteurii is a nickel-binding GTPase which assists the folding of the enzyme urease [32]. Site directed mutagenesis was applied to mutate the unique natural occurring cysteine (Cys68) to alanine (Cys68Ala) and to introduce a new cysteine residue at position 158 (Asp158Cys). The protein was purified as already described [33]. The spin labeling procedure was as follows: Prior to spin-labeling, TCEP (from purification step) was removed by desalting with PD10 desalting column (GE Healthcare, Chicago, IL, USA) using Tris-HCl 10 mM pH 7.4 as elution buffer. The spin labeling reaction was carried out by incubating 100 nmoles of protein with a 10-fold molar excess of Maleimido-proxyl nitroxide (Sigma-Aldrich, St. Louis, MO, USA). A second PD10 desalting column against the same buffer was performed to remove the unbound spin-label. The labeled protein was concentrated by centrifugation in 2 mL Vivaspin concentrators 3 kDa MWCO.

### 3.2. EPR Acquisitions

X band EPR spectra (RT and LT) were recorded on an Elexsys E500 spectrometer (Bruker Corporation, Billerica, MA, USA) equipped with a variable temperature accessory and a super high sensitivity resonator operating at 9.5 GHz. Low temperatures (115K) were achieved with liquid nitrogen using a Bruker variable temperature unit (ER4101VT). At room temperature, the microwave power and the magnetic field modulation amplitude were 10 mW and 0.1 mT, respectively. At 115K, the microwave power and the magnetic field modulation amplitude were 0.4 mW and 0.4 mT, respectively. The magnetic field modulation frequency was 100 kHz.

### 3.3. Calibration of the Magnetic Field of the EPR Spectra

For X-band spectra, the magnetic field was calibrated for each sample, using as a reference the g value of the ER4119HS-2100 marker accessory (g = 1.979896) (Bruker Corporation, Billerica, MA, USA). To avoid the spectrum to be distorted by the signal of this marker, two spectra were recorded consecutively, one with the g-marker in the resonator for the field calibration and one with the g-marker out for the simulation. Practically, the “in” spectrum was subtracted from the “out” spectrum, resulting in a g-marker alone spectrum in the “out” conditions. The magnetic field offset obtained for this spectrum was applied to the “out” spectrum to be simulated. If no g-marker is available, DPPH or other standard of known g-value can be used. In this case, it is recommended to record the spectrum for the field calibration just before or after recording the spectrum to be simulated.

### 3.4. Simulation and Automatic Fitting

All the simulations reported here were run with *SimLabel*, most of the time in *real time* mode to instantaneously appreciate the effect on the spectrum of the variation of a given parameter. The *EasySpin* function used to simulate these spectra recorded at room temperature was *chili*, the *slow motion* mode in *SimLabel*. In *SimLabel*, *chili* is used with the default settings and no ordering potential.

The automatic fittings were performed with the *esfit* GUI of *EasySpin* launched from *SimLabel*. This GUI enables to fit simulated EPR spectra to experimental spectra using least-squares fitting algorithms. Each automatic fitting took place in two steps: a global automatic fitting followed by a local automatic fitting. With a maximum of five parameters, the global fitting was achieved with the *grid search* method otherwise the *genetic algorithm* was used. The local fitting was performed with the *Nelder/Mead simplex* method. The *Target* and *Scaling* were always *data as is* and *scale & shift (lsq0)*, respectively. The *Startpoint* was alternatively *center of range* or *selected parameter set*. Particular attention was paid to parameters reaching the limit of their range of variation. In such a case, a new fitting (global or local) was run with a new range of variation (same width, new center).

## 4. Conclusions

The simulation of X-band cw-EPR spectra of nitroxide labeled proteins at room temperature enables to quantify dynamics and to obtain biologically relevant information. *Simlabel* provides an easy way to perform such simulations. However, the high number of available parameters makes their unambiguous determination very difficult.

The systematic review of the parameters used in *SimLabel* and the effect of their variations in controlled ranges has led us to propose guidelines to perform such simulations. Keeping in mind the necessity to decrease as much as possible the number of adjustable parameters, we propose to set g_y_ and g_z_, to reduce the range of variations of A_xy_, whatever the considered case, and to assign A_z_ and g_x_ before starting the fitting procedure. Thanks to these considerations, by slowly increasing the complexity of the model, a simulation close to the experimental spectrum should be obtained in a few minutes.

Thus, these step-by-step guidelines applied to a given experimental spectrum should help different users to run their simulations in a satisfactory and reproducible manner. If applied by the majority of users, this should allow comparing the parameters obtained by the simulation from one study to another.

## Figures and Tables

**Table 1:** Variable parameters used to obtain the simulations shown in magenta Figure 5b–h for sample 1. The underlined values indicate the modified parameters from the previous simulation. The other fixed parameters are: A_z_ = 3.64 mT, g_x_ = f(A_z_) = 2.0084, g_y_ = 2.0061 and g_z_ = 2.0022 at 9.45 GHz.

	(b)	(c)	(d)	(e)	(f) ^2^	(g) ^2^	(h) ^2^
t_corr_ (ns)	0.50	0.50	1.0	0.65	0.53	1.6 (x,z)	1.0 (x,z)
						0.26 (y)	0.33 (y)
A_xy_ (mT)	0.50	0.57	0.57	0.57	0.57	0.57	0.57
lw_lor_ (mT)	0	0	0	0.10	0.13	0.10	0.12
*Fit indicator* ^1^	0.452	0.345	0.031	0.016	0.017	0.014	0.008

^1^ The *Fit indicator* values shown here were calculated in *SimLabel* with the derivative line. ^2^ The correlation time is axial. (x, z) and (y) indicate the considered direction(s).

**Table 2:** Variable parameters used to obtain the simulations shown in magenta Figure 6b–f for sample 2. The fast component and the slow component refer to the blue and green traces shown Figure 6, respectively. The other fixed parameters are: Az = 3.54 mT, g_x_ = f(A_z_) = 2.0087, g_y_ = 2.0061 and g_z_ = 2.0022 at 9.45 GHz.

		(b)	(c)	(d)	(e) ^2^	(f) ^2^
fast	t_corr_ (ns)	0.50	0.56	0.53	1.8 (x,z)	1.5 (x,z)
					0.22 (y)	0.26 (y)
	weight (%)	100	25	26	29	29
slow	t_corr_ (ns)	-	8.3	9.3	8.3	9.3
	weight (%)	-	75	74	71	71
Slow & fast	A_xy_ (mT)	0.60	0.60	0.62	0.60	0.61
	lw_lor_ (mT)	0	0.07	0.15	0.10	0.12
*fit indicator* ^1^		0.538	0.083	0.033	0.015	0.012

^1^ The *Fit indicator* values shown here were calculated in *SimLabel* with the derivative line. ^2^ The correlation time of the fast component is axial. (x,z) and (y) indicate the considered direction(s).

## Data Availability

Not applicable.
